# Nesprin proteins: bridging nuclear envelope dynamics to muscular dysfunction

**DOI:** 10.1186/s12964-024-01593-y

**Published:** 2024-04-02

**Authors:** Zhou Zi-yi, Qin Qin, Zhou Fei, Cao Cun-Yu, Teng Lin

**Affiliations:** 1 Department of Cardiology, Yichang Central People’s Hospital, Yichang, 443003 Hubei People’s Republic of China; 2 School of Basic Medicine, China Three Gorges University, Yichang, 443000 Hubei People’s Republic of China; 3 King’s College London British Heart Foundation Centre of Research Excellence, School of Cardiovascular and Metabolic Medicine & Sciences, London, SE5 9NU UK; 4 College of Basic Medical Sciences, Hubei Key Laboratory of Tumor Microencironment and immunotherapy, China Three Gorges University, Yichang, 443000 Hubei People’s Republic of China

**Keywords:** LINC complex, Nesprin, Muscular diseases, Nuclear-cytoskeletal interactions, Molecular mechanisms, Therapeutic interventions

## Abstract

This review presents a comprehensive exploration of the pivotal role played by the Linker of Nucleoskeleton and Cytoskeleton (LINC) complex, with a particular focus on Nesprin proteins, in cellular mechanics and the pathogenesis of muscular diseases. Distinguishing itself from prior works, the analysis delves deeply into the intricate interplay of the LINC complex, emphasizing its indispensable contribution to maintaining cellular structural integrity, especially in mechanically sensitive tissues such as cardiac and striated muscles. Additionally, the significant association between mutations in Nesprin proteins and the onset of Dilated Cardiomyopathy (DCM) and Emery-Dreifuss Muscular Dystrophy (EDMD) is highlighted, underscoring their pivotal role in disease pathogenesis. Through a comprehensive examination of DCM and EDMD cases, the review elucidates the disruptions in the LINC complex, nuclear morphology alterations, and muscular developmental disorders, thus emphasizing the essential function of an intact LINC complex in preserving muscle physiological functions. Moreover, the review provides novel insights into the implications of Nesprin mutations for cellular dynamics in the pathogenesis of muscular diseases, particularly in maintaining cardiac structural and functional integrity. Furthermore, advanced therapeutic strategies, including rectifying Nesprin gene mutations, controlling Nesprin protein expression, enhancing LINC complex functionality, and augmenting cardiac muscle cell function are proposed. By shedding light on the intricate molecular mechanisms underlying nuclear-cytoskeletal interactions, the review lays the groundwork for future research and therapeutic interventions aimed at addressing genetic muscle disorders.

**Supplementary Information:**

The online version contains supplementary material available at 10.1186/s12964-024-01593-y.

## Introduction

The nucleus, a central organelle within the cell, orchestrates subcellular organization beyond its traditional role as a repository of genetic material [1, 2]. Its dynamic interplay with diverse organelles is facilitated by the cytoskeleton, establishing critical connections crucial for spatial organization [3–5]. While epithelial cells typically position the nucleus proximal to the basement membrane via microtubule-driven mechanisms, fibroblasts rely on actin filaments for nuclear localization [6, 7]. Remarkably, in skeletal and cardiac muscle cells, the nucleus acts as a Microtubule Organizing Center (MTOC), significantly impacting cellular architecture and microtubule arrangement during muscle development. Mechanisms governing nuclear positioning can be classified into two distinct types [8, 9]. The first operates independently of direct cytoskeleton-nuclear envelope interactions, potentially restricting specific cytoplasmic elements’ access to the perinuclear region. This mechanism indirectly influences nuclear positioning by modulating local fiber density or inducing movement of other cytoplasmic components [10]. Conversely, the second mechanism relies on the physical coupling between the cytoskeleton and the nuclear envelope, exerting direct forces on the nucleus. The presence of the LINC complex further complicates this physical linkage [11, 12]. Responsible for establishing a stable yet dynamic connection between the nuclear membrane and cytoskeleton, the LINC complex plays a pivotal role in transmitting forces and signals crucial for nuclear shaping, positioning, and centrosome-nucleus association. Its significance extends to fundamental cellular processes, including DNA repair, nuclear envelope organization, cell migration, and chromosomal dynamics during meiosis [13].

The LINC complex stands as a cornerstone in cellular mechanics, orchestrating essential connections between the nuclear envelope and the cytoskeleton. Particularly, Nesprin proteins, integral components of the LINC complex, emerge as pivotal mediators in this intricate interplay, influencing cellular morphology, structure, and mechanical stability [14]. Understanding their role is paramount, especially in mechanically sensitive tissues like cardiac and striated muscles, where disruptions in the LINC complex are implicated in various muscular disorders [15, 16]. This review delves into the multifaceted functions of Nesprin proteins within the LINC complex, elucidating their involvement in muscular diseases such as DCM and EDMD, and exploring novel therapeutic strategies aimed at rectifying Nesprin-related pathologies. Through a comprehensive examination, we aim to shed light on the intricate molecular mechanisms underlying nuclear-cytoskeletal interactions and pave the way for innovative therapeutic interventions in genetic muscle disorders.

## Role of the LINC complex in cellular mechanics and muscle function

**Fig. 1:**
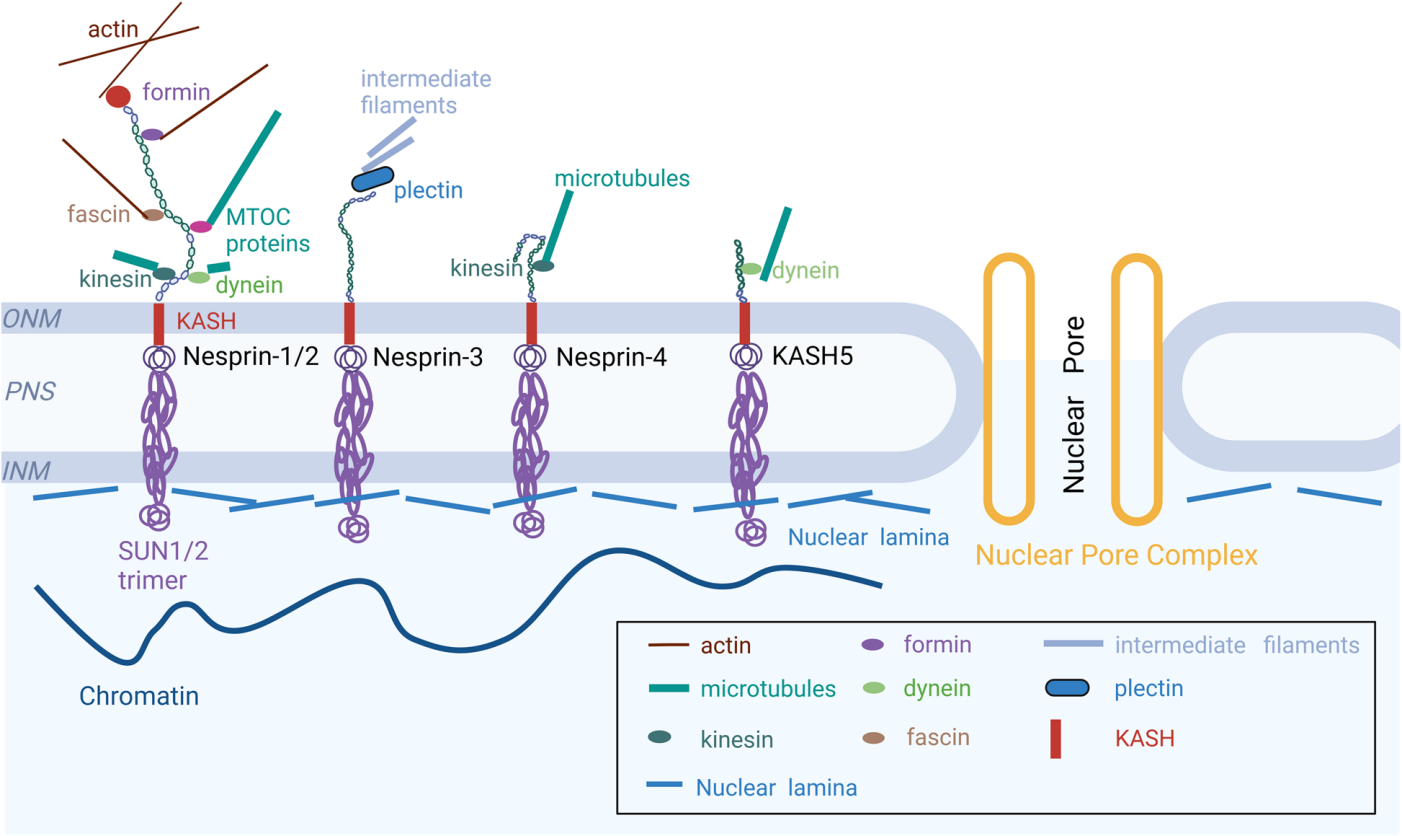
The cytoskeletal interactions of the LINC Complex and Nesprin at the Nuclear Envelope. Giant Nesprin-1/2 localizes to the nuclear membrane and interacts with SUN-1/2, facilitating the formation of the LINC complex, which physically connects the cytoskeleton and the nucleus. The KASH domain of Nesprins, located at the outer nuclear membrane (ONM), interacts with the SUN domain situated in the perinuclear space (PNS), found between the ONM and inner nuclear membrane (INM). Each Nesprin isoform (Nesprin-1, Nesprin-2, Nesprin-3, Nesprin-4, and KASH5) exhibits specific positioning, forming distinct interactions with SUN proteins and displaying unique affinities for various cytoskeletal components. Nesprin-1/2 interact with SUN proteins in the perinuclear space and associate with actin filaments, playing a critical role in linking the nucleus to the actin cytoskeleton. Nesprin-3, located at the ONM, interacts with SUN proteins and exhibits a specific association with intermediate filaments, facilitating the connection between the nucleus and intermediate filament networks. Nesprin-4 interacts with SUN proteins in the perinuclear space and demonstrates an affinity for microtubules, contributing to tethering the nucleus to the microtubule cytoskeleton. KASH5, exclusive to meiotic cells, acts as an activator adapter for motor proteins, revealing the hierarchical assembly of the KASH5-motor protein-dynein complex. Nesprin-4 and KASH5 interact with microtubule motor proteins, including kinesin-1 or dynein/dynactin complexes

**Fig. 2:**
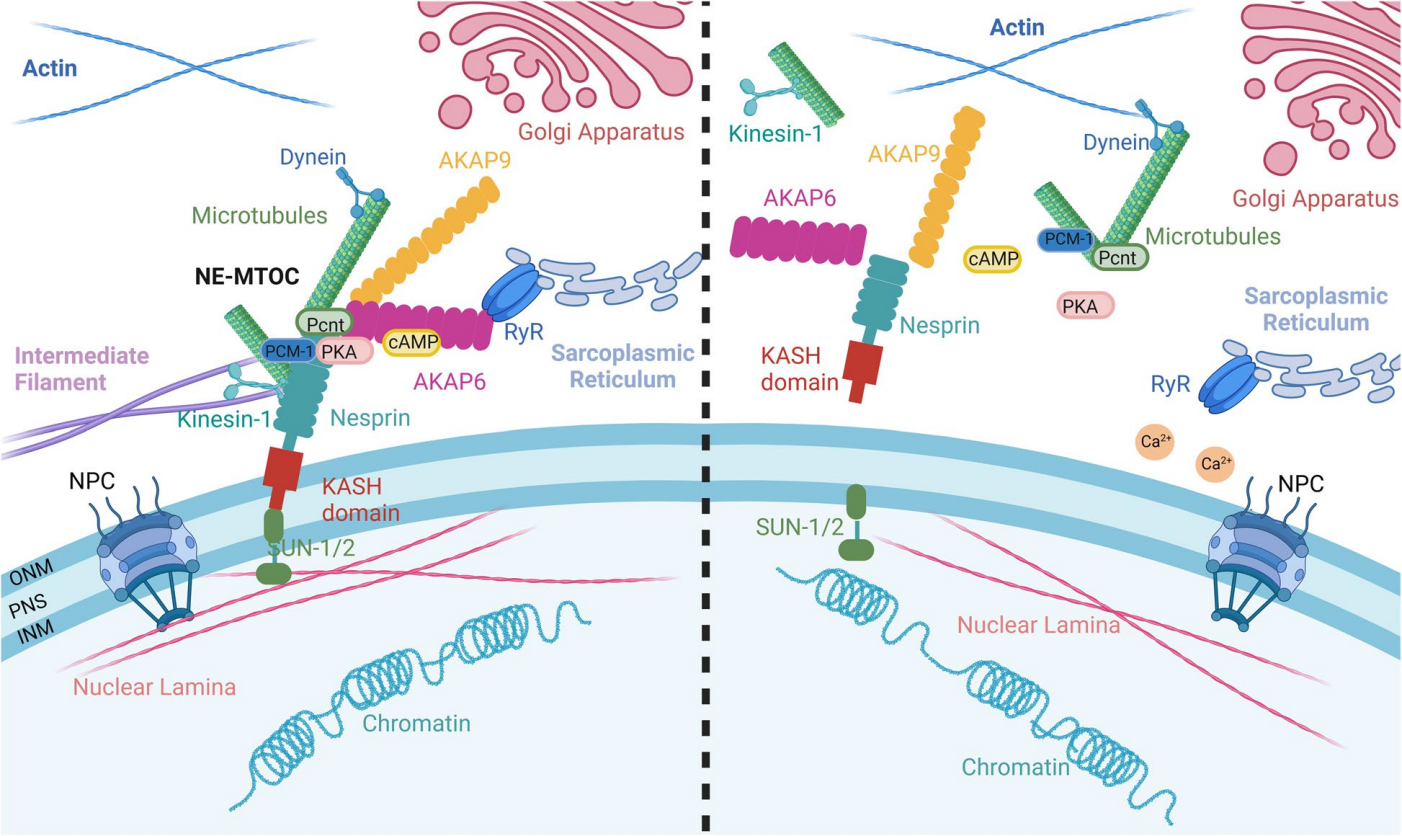
The Role of Nesprin in LINC Complex Formation and Nuclear-Cytoskeletal Connectivity. **A** Nesprin plays a pivotal role in facilitating the formation of the LINC complex and establishing connectivity between the nucleus and the cytoskeleton. Nesprin proteins interact with SUN domain proteins, facilitating the physical connection between the nucleus and the cytoskeleton and enabling crucial cellular processes such as mechanotransduction, nuclear positioning, and intracellular signaling. Through its various isoforms and domain structures, Nesprin exhibits specific affinities for different cytoskeletal components, including actin filaments, intermediate filaments, and microtubules, further contributing to the versatility and functionality of the LINC complex in maintaining cellular integrity and homeostasis. Additionally, the LINC complex mediates chromatin binding through interactions with the nuclear lamina. Giant Nesprin-1/2 directly interfaces the nucleus with the actin cytoskeleton, while shorter Nesprin isoforms like Nesprin-1α_2_ associate with microtubules via KLC-1/2 and AKAP6. AKAP6, in turn, tethers centrosomal proteins such as PCM-1 and AKAP9 to the nuclear membrane, forming a cAMP-PKA signaling hub that may regulate nuclear envelope calcium dynamics through interactions with RyR on the sarcoplasmic reticulum. **B** Mutations in Nesprin can disrupt the LINC complex, uncoupling the cytoskeleton from the nucleus and inducing structural aberrations such as abnormal nuclear morphology, size, migration, or positioning. This perturbation can trigger the activation of signaling cascades and mechanosensitive transcription factors, ultimately resulting in cellular dysfunction

The LINC complex stands as a pivotal mediator of mechanical coupling between the nuclear membrane and the cytoskeleton, crucial for sensing and regulating mechanical stimuli while maintaining cellular morphology, structure, and mechanical stability [14, 17]. Composed of SUN (Sad1/UNC84) and KASH (Klarsicht/ANC-1/Syne) proteins, the mammalian genome encodes at least five SUN proteins (SUN1-5) and six KASH proteins (Nesprin1-4, KASH5, LRMP), forming the core constituents of the LINC complex. These proteins establish stable connections between the nuclear membrane and the cytoskeleton, facilitating mechanical transmission between the nucleus and the cytoskeleton, thereby influencing cellular polarity, migration, mechanosensing, and gene expression [18] (Fig. 1). The adapter proteins within the LINC complex, including Nesprin, SUN, and lamins, exhibit abundant expression in cardiac and skeletal muscles. Nesprin, harboring a KASH domain within the outer nuclear membrane (ONM), directly interfaces with the cytoskeleton (including microtubules, actin, and intermediate filaments) in the cytoplasm. Concurrently, Nesprin interacts with SUN proteins, anchoring itself to the dense nuclear lamina beneath the inner nuclear membrane (INM) [19]. This intricate network of physical connections among the cytoskeleton, LINC complex, and nuclear lamina establishes a robust structural foundation for the direct transmission of extracellular forces into the cell nucleus, thereby allowing mechanical signals to modulate gene transcription [17] (Fig. 2A).

The importance of the LINC complex extends across diverse model organisms [20–22]. Studies in *Caenorhabditis elegans* have elucidated the critical function of actin-associated nucleus component 1 (ANC-1), a homolog of Nesprin-1/2, in actin-mediated nuclear positioning within muscle cells. Conversely, mutations in the SUN1 homolog UNC-84 disrupt both nuclear migration and anchorage [23]. Further investigations in mice and human fibroblasts have unveiled that abnormalities in the LINC complex or the absence of specific Nesprin/SUN proteins lead to defects in centrosome attachment to the nucleus. Notably, cells lacking the LINC complex exhibit impaired polarization responses in wound healing assays, thereby compromising nuclear repositioning and centrosome movement [24]. Moreover, endothelial cells with reduced Nesprin-1 struggle to reorient under cyclic strain, consequently impacting cell migration. Meanwhile, Nesprin-2 plays a crucial role in facilitating rearward nuclear movement in fibroblasts, a process preceding polarization and migration [25]. Disruption of the LINC complex poses a significant hurdle to cell polarization, with fibroblasts harboring defects in nuclear lamin A/C struggling with nuclear polarization and migration [26]. Engineered mice with genetic mutations in the LINC complex or lamin manifest aberrant synaptic nuclear positioning in skeletal muscle neuromuscular junctions [15, 27]. These findings address a fundamental inquiry: How do nuclear membrane proteins influence cellular structure and functionality? The orchestrated movement of nuclei during cell migration corresponds with cytoskeletal remodeling, ensuring the nucleus’s stability amidst a dynamic cellular milieu. Synaptic nuclei migration toward the cell periphery in muscle cells necessitates effective cytoskeletal forces to maneuver the nucleus. Firm anchorage to the cytoskeleton proves pivotal in maintaining nucleus stability and averting random displacement [28–30]. Pioneering studies employing controlled mechanical forces on adherent fibroblasts have revealed the intricate mechanism of force transmission within cells. Disrupted LINC complexes exhibit diminished nuclear displacement under mechanical strain, underscoring the pivotal role of this complex in transmitting forces between the nucleus and cytoskeleton [31, 32]. Such transmission significantly impacts cell migration and polarization by regulating nuclear positioning and movement. The integrity of the LINC complex emerges as crucial for maintaining cellular mechanical stability and facilitating adaptive responses to environmental stimuli [33]. A deeper understanding of these mechanisms offers insights into intracellular mechanical signal transduction and unveils potential therapeutic strategies for associated diseases.

The significance of the LINC complex is particularly pronounced in mechanically active tissues like striated muscles, where it acts as a crucial link connecting the cytoskeleton and contractile mechanisms with the nucleus. In vitro experiments have shed light on the essential role of microtubules in preserving nuclear shape, thereby contributing significantly to nuclear integrity and structure [34]. Investigations in rat cardiomyocytes, where desmin or its associated Nesprin-3 were deleted, revealed microtubule-dependent nuclear invagination, leading to DNA damage, disrupted nuclear envelope-chromatin interactions, and transcriptomic alterations. Co-expression of dominant-negative *KASH* genes mitigated nuclear invagination, suggesting an interaction between microtubules and Nesprin-1 and/or Nesprin-2 in this process [35, 36]. Intriguingly, Nesprin-1 Calponin homology (CH) domains Knockout (KO) mice lacking the actin-binding domain of Nesprin-1G exhibited survival without apparent striated muscle defects, suggesting a potentially non-essential role of Nesprin-1G and its homologs in muscle structure and function [37]. Moreover, in lamin A/C gene (*LMNA*) knockout muscle fibers, disruption of the LINC complex resulted in increased cell viability and contractility, highlighting the role of Nesprin-1 in transmitting microtubule-mediated mechanical tension to the nucleus [32]. These findings underscore the paramount importance of the LINC complex in preserving cellular structural integrity and function, particularly in mechanically sensitive tissues such as cardiac and striated muscles. They accentuate the significance of the microtubule network, Nesprin proteins, and other cytoskeletal components in maintaining nuclear shape and preventing damage. Forming a sophisticated molecular network beneath the nuclear envelope, the LINC complex, connected with nuclear membrane protein emerin and the nuclear lamina, facilitates mechanical force transfer and direct regulation from the cytoskeleton to the nuclear genome, highlighting its crucial role in transmitting mechanical signals [15, 38, 39]. Mutations in LINC complex components have been implicated in various nuclear envelope diseases in muscle physiology, including DCM and EDMD, among other striated muscle disorders. Disruption of the LINC complex due to gene mutations leads to structural defects, chromatin disorganization, abnormal nuclear morphology, myonuclear positioning, impaired force transmission, and altered gene regulation, all contributing to muscle diseases (Fig. 2B). The integrity of the LINC complex is vital for maintaining nuclear structure and function, underscoring its significance in cell development and function [15, 25, 27, 35, 40, 41]. Understanding the LINC complex and its components not only elucidates muscle disease mechanisms but also unveils potential treatments for nuclear envelope disorders and related diseases.

## Nesprin protein variants: diverse functions and tissue localization

Nesprin-1 and Nesprin-2, classified as ‘giant’ isoforms, stand out as some of the largest proteins known, boasting impressive molecular weights of 1.01 MDa and 796 kDa, respectively. Spanning 146 and 116 exons, respectively, these proteins share a remarkable 64% structural homology, emphasizing their close relationship and functional parallels [15, 42, 43]. Originally identified as markers for vascular smooth muscle cell differentiation and synaptic muscle components, subsequent research has unveiled their extensive expression across diverse cell types, underscoring their profound biological significance [15, 42, 44–46]. Located at the outer nuclear membrane, Nesprin-1 and − 2, as formidable proteins, consist of three primary structural domains. The N-terminal region harbors paired CH domains crucial for actin interactions. The central domain, comprising multiple spectrin repeats (SRs), serves as a scaffold connecting the CH and KASH domains, facilitating various protein interactions. The C-terminal KASH domain interacts with proteins on the INM containing the SUN domain. Of particular note is the highly conserved adaptive domain (AD) at the C-terminus of Nesprin-1 and − 2, which plays a pivotal role, particularly in stabilizing the conformation of the SRs [15, 42, 47]. Nesprin-3, the third member of the Nesprin family, interacts with members of the plakin family, particularly binding to the intermediate filament cell junction protein, plectin, to maintain its widespread presence. Overexpression of Nesprin-3 leads to significant recruitment of plectin to the nuclear periphery, where both molecules colocalize with keratin-6 and − 14. Plectin forms connections with integrin α_6_β_4_ on the cell surface and Nesprin-3 on the ONM in keratinocytes, indicating a continuous link between the nucleus and the extracellular matrix through the intermediate filament cytoskeleton [46]. In acephalic sperm syndrome, SUN5 plays a crucial role in positioning Nesprin-3 to the posterior nuclear membrane, essential for connecting the sperm head and tail [48]. Recent studies have highlighted the pathological role of sperm-associated antigen 4 (SPAG4) in cooperation with Nesprin-3 in lung cancer cell migration [49]. Nesprin-4, primarily expressed in epithelial cells, correlates with significant changes in cell organization, including the repositioning of the centrosome and Golgi apparatus relative to the cell nucleus [50]. Defects in Nesprin-4 result in mispositioning of outer hair cell (OHC) nuclei and cell death, leading to deafness in humans and mice. LRMP, enriched in mammalian taste receptor cells and a subset of *zebrafish* fertilized eggs, binds to the calcium channel IP3 receptor, controlling taste signal transduction [51–53]. KASH5, restricted to meiotic cells, serves as an activator adapter for motor proteins, revealing the hierarchical assembly of the KASH5-motor protein-dynein complex. Nesprin-4 and KASH5 interact with microtubule motor proteins, kinesin-1 or dynein/dynactin complexes [54] (Fig. 1). Nesprin proteins engage with cytoskeletal elements such as actin, microtubules (MT), and intermediate filaments (IFs) via their N-terminus, contributing to the formation of the cytoplasmic cytoskeletal network and tethering extranuclear molecules to the ONM [23, 55, 56]. The broad functional scope of Nesprins becomes apparent through their interactions with various linkers of the LINC complex, strengthening the bond between the nucleus and filamentous cytoskeletal networks.

**Fig. 3:**
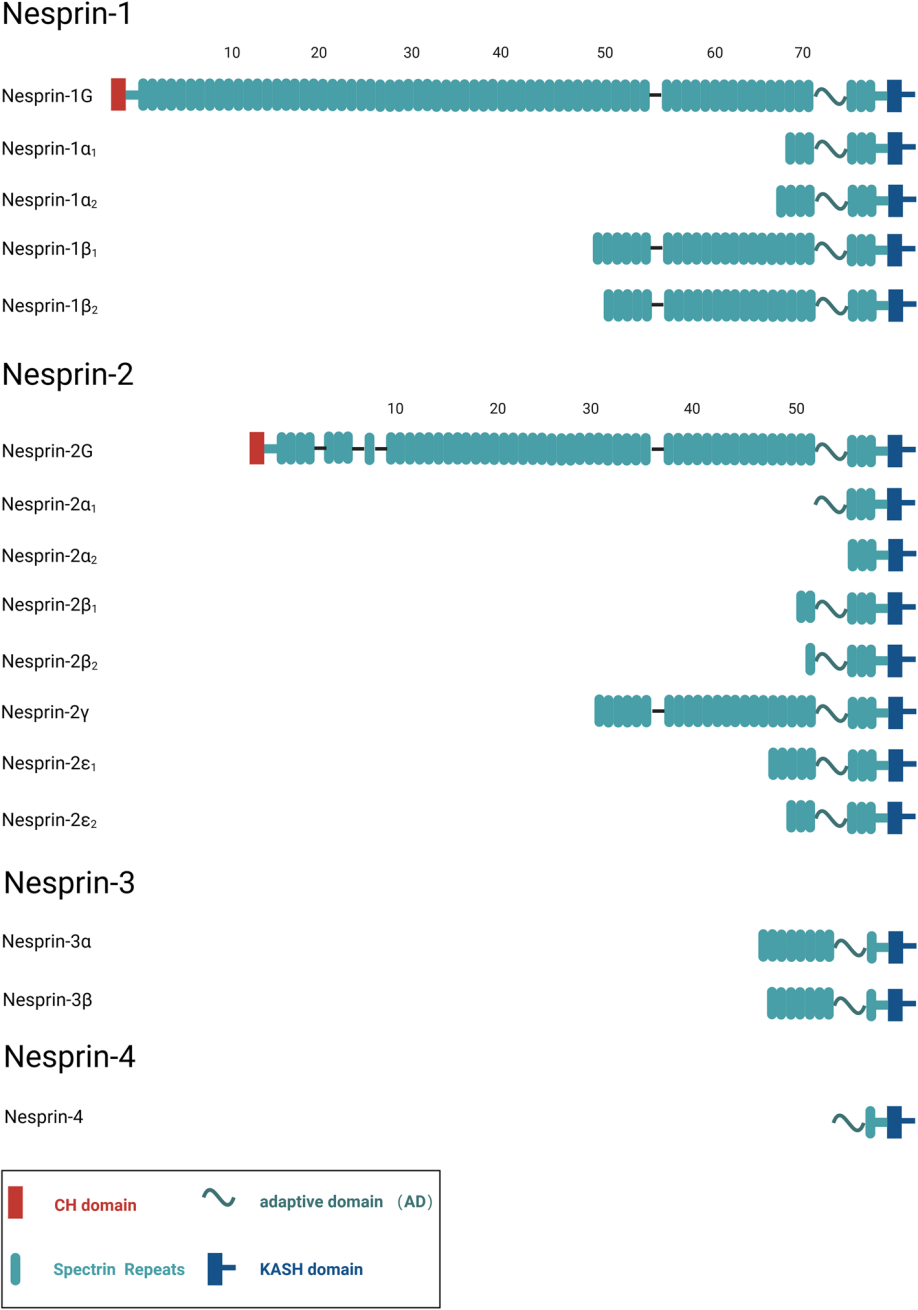
Structure of Giant Nesprins and Isoforms. The structure of giant Nesprin-1/2 comprises three primary domains: tandem actin-binding CH domains, a central rod consisting of spectrin repeats (SRs), and the NE-targeting KASH domain, which includes a transmembrane (TM) domain and a luminal KASH peptide. Additionally, at the C-terminus, there is a highly conserved adaptive domain (AD) crucial for structurally stabilizing the SRs. Various Nesprin isoforms exist, some of which are depicted above, each with SRs close to the C-terminus. Nesprin-3 consists of a KASH domain and 7 SRs(3β) or 8SRs(3α).Nesprin-4 is the smallest member of the Nesprin family. It contains 1 SRs protein sequence and lacks an actin-binding domain (ABD).However, these isoforms often lack critical structural domains (e.g., CH or SRs) and display distinct expression patterns and localizations across diverse tissues

 Nesprin-1 and Nesprin-2, encoded by *SYNE1* and *SYNE2* genes respectively, exhibit extensive isoform diversity resulting from transcriptional processes, termination, and alternative splicing. These isoforms, integral to various cellular functions, often lack critical structural domains (e.g., CH, KASH, or SRs) and display distinct expression patterns and localizations across diverse tissues [57] (Fig. 3, Supplementary data Table 1). For example, Nesprin-1α_2_, present in both the ONM and INM, serves as a notable illustration of this diversity. Other Nesprin-1 and Nesprin-2 isoforms localize to various cellular compartments, including the Z-lines of sarcomeres in skeletal and cardiac muscles, focal adhesion sites, Golgi apparatus, and actin stress fibers [58, 59]. Particularly relevant to cardiac and skeletal muscles are highly expressed isoforms such as Nesprin-1α_2_, Nesprin-2α_1_, and Nesprin-2ε_2_, which primarily consist of evolutionarily conserved regions, including the C-terminal SRs and adjacent unstructured ADs [60–65]. These regions serve as binding sites for various interacting proteins, including lamin A/C and emerin. Among these isoforms, Nesprin-1α_2_, a smaller variant of Nesprin-1 weighing approximately 112 kDa, comprises the last six SRs of the giant Nesprin-1 protein’s C-terminal region and features a distinct N-terminal region composed of 31 amino acids, originating from a unique initiation site [60, 64]. During the process of muscle differentiation, Nesprin-1α_2_ exhibits prominent localization at the ONM, accompanied by a substantial increase in expression levels. This isoform interacts with pivotal constituents of the outer nuclear MTOC, including kinesin-1 subunits, kinesin light chain-1/2 (KLC-1/2), and alpha-kinase anchoring protein 6 (AKAP6). Such interaction with perinuclear microtubules reveals a novel role for Nesprin-1α_2_ in the function of striated muscles. Notably, pathogenic mutations linked to striated muscle diseases are predominantly located in the C-terminal region of Nesprin, particularly affecting Nesprin-1α_2_.This observation underscores the paramount importance of this region in the physiology of striated muscles. Insights derived from these studies carry significant implications for understanding the potential involvement of Nesprin-1α_2_ in muscle pathologies, potentially laying the groundwork for novel therapeutic interventions targeting these diseases [15, 27, 66, 67] (Fig. 2).

## The spectrum of disorders associated with Nesprin dysregulation

To date, mutations in *SYNE1/2* have been identified through the screening of patients with muscle-specific disorders, including EDMD and DCM, comprising over 3.5% of causative mutations [27]. The mutations identified in the C-terminus of Nesprin-1/2 have been extensively characterized, demonstrating their capacity to induce defects in nuclear morphology, aberrant localization, and disrupted binding of SUN1/2, lamin A/C, and emerin, thereby impairing myogenesis. These disruptions in the LINC complex mirror those observed with mutations in lamin A/C and emerin. Furthermore, cardiac muscle phenotypes are evident in two *SYNE1* and/or *SYNE2* knockout mouse models, both of which display disrupted LINC complexes [25, 32, 68].

### DCM

DCM represents a critical condition characterized by impaired left ventricular expansion and contraction, often culminating in heart failure and sudden cardiac death, particularly among younger individuals [69]. Recent investigations have unveiled that mutations in the *LMNA* gene, responsible for encoding lamin A/C, account for up to 5% of familial DCM cases. Understanding how mutations in lamin A/C and emerin, proteins ubiquitously expressed across diverse tissues, contribute to muscle-specific disorders has emerged as a central focus of scientific inquiry [70]. Research underscores the pivotal role of their interacting partners—Nesprin-1, Nesprin-2, and SUN1/2—in the progression of these diseases. Cellular studies involving DCM patients have unveiled mutations often leading to altered nuclear morphology. Fibroblasts or lymphocytes samples frequently exhibit nuclear abnormalities, including wrinkling, micronuclei formation, swelling, and fragmentation. Cells afflicted by DCM commonly show notable disturbances in the localization of LINC complex components and changes in the binding of Nesprin-1 and − 2. These mutations substantially disrupt the interconnections among Nesprin, lamin A/C, SUN proteins, and the microtubule motor protein KLC-1/2 [25, 27]. Investigations on Nesprin-1 mutated C2C12 cells associated with DCM have revealed reduced fusion capacity, decreased expression of myogenic proteins, and diminished MHC expression during myocyte differentiation. Moreover, heightened levels of Nesprin-1 were detected in fibroblasts from these patients [27]. Reports underscore disruptions in the LINC complex, alterations in nuclear morphology, and disorders in muscular development in DCM cases linked to mutations in lamin A/C or emerin, emphasizing the crucial role of an intact LINC complex in preserving muscle physiological functions [71]. These findings not only advance our comprehension of DCM’s pathophysiology but also identify potential therapeutic targets within these specific molecular pathways.

### EDMD

EDMD manifests as a rare genetic myopathy encompassing a spectrum of potentially life-threatening cardiac complications, necessitating prompt and precise diagnosis. Clinical presentations include muscle weakness, early-onset muscle contractures, cardiac conduction anomalies, and cardiomyopathy, exhibiting significant variability across EDMD subtypes and individual cases [72]. The genetic landscape of EDMD encompasses genes such as *EMD*, *LMNA*, *SYNE1*, *SYNE2*, *FHL1*, *TMEM43*, *SUN1*, *SUN2*, and *TTN*, encoding emerin, lamin A/C, Nesprin-1, Nesprin-2, FHL1, LUMA, SUN1, SUN2, and Titin, respectively. However, more than 60% of EDMD patients do not harbor mutations in these established genes (*EMD* or *LMNA*), implying the existence of unidentified pathogenic genes [25, 56, 70, 73–90].

EDMD is characterized by a triad of symptoms, with retraction appearing first, followed by muscle weakness and wasting, and finally, the development of cardiac dilation [72]. Cardiac complications, such as atrial tachycardia, atrial standstill, ventricular tachycardia, and cardiomyopathy, are prevalent in EDMD. Symptoms typically manifest in the second decade of life, including palpitations, pre-syncope and syncope, exercise intolerance, and heart failure. Notably, skeletal muscle weakness typically precedes cardiac symptoms in EDMD [91, 92]. However, it’s important to clarify that the elevated risk of cardiac complications in female EDMD patients primarily applies to cases caused by mutations in X-linked genes such as *EMD* and *FHL1*. In these cases, females serve as carriers but may not necessarily develop symptoms or may experience less severe symptoms [74]. Moreover, EDMD patients with *LMNA* mutations beyond 50 years of age exhibit over a 60% incidence of heart failure, underscoring the necessity for deeper insights into EDMD’s genetic underpinnings and pathomechanisms to enhance diagnostic accuracy, develop targeted therapies, and improve patient quality of life [93].

The pathogenesis of EDMD involves mutations in various genes, including the *TMEM43* gene, encoding the nuclear envelope protein LUMA. LUMA plays a pivotal role in nuclear envelope structuring and maintenance, collaborating with emerin and lamin A/C to preserve nuclear integrity and functionality [94]. Mutations in *LUMA* result in atypical nuclear shapes, impacting cellular functions. LUMA is crucial for emerin positioning, and its interaction with SUN2 protein is critical; mutated *LUMA* may disrupt SUN2 intranuclear localization by binding to it, contributing to its dysfunction [83, 94]. Additionally, altered Nesprin presence can affect emerin and lamin proteins’ positioning, while mutant lamin A proteins can lead to the mislocalization of LAP2, Nup153, and lamin B. Disrupted interactions between mutated SUN1, lamin A/C, and emerin could result in nuclear functional anomalies. Skeletal muscle biopsies of EDMD patients often exhibit clustered nuclei, increased variability in fiber size, fibrosis, and significant adipose tissue presence [41, 95].

Recent discoveries have unveiled multiple mutations in Nesprin-1, Nesprin-2, and SUN1/2 in both DCM and EDMD patients. Mislocalization of lamin A/C and SUN2 has been observed in fibroblasts from EDMD patients and in neonatal rat cardiomyocytes transfected with Nesprin-1 isoforms carrying DCM-related mutations. These gene mutations, considered independent pathogenic agents for muscle diseases, include SUN1/2, which are viewed as disease-modifying genes for other causative genes of EDMD, such as *LMNA*, *EMD*, and *LAP2α* [16, 27].Nesprin mutations variably impact LINC complex functions, particularly in the C-terminal SR region, inducing nuclear morphology defects and disrupting binding with nuclear membrane-associated proteins. Such disruptions lead to LINC complex instability, compromising mechanical transduction within muscle cells, critical under mechanical strain [14, 17]. Mutations in the AD region of Nesprin significantly affect its interaction with microtubule motor proteins KLC-1/2, potentially leading to defects in nuclear migration and positioning, impeding muscle development and maturation [25]. This intricate network of interactions among nuclear envelope proteins underscores their pivotal role in maintaining nuclear structure and cellular functionality, particularly in tissues subjected to significant mechanical stress like cardiac and skeletal muscles.

## Deciphering Nesprin mutations and their implications for cellular dynamics in muscular disease pathogenesis

In the realm of muscular disease pathologies, Nesprin-1/2 plays a crucial role by localizing at the nuclear envelope and forming the LINC complex alongside SUN1/2, lamin A/C, and emerin. This complex acts as a vital bridge between the nucleus and the actin cytoskeleton [37, 96]. Ongoing investigations into these diseases propose two significant hypotheses. The structural hypothesis emphasizes the role of the LINC complex in tethering the nuclear scaffold to the cytoskeleton and associated proteins, including molecular motors, microtubule-associated proteins (MAPs), microtubules, and the actin network. Disruption of this complex can compromise extracellular forces, rendering cells susceptible to mechanical damage [72, 96–99].The pivotal role of Nesprin in myonuclear positioning spans organisms from *Caenorhabditis elegans* to higher ones, where mutations impede the recruitment of partners like Akap450 or KLC-1/2, resulting in misplacement of nuclei [72, 97–99]. The gene regulation hypothesis suggests that compromised LINC complexes may alter nuclear membrane protein interactions with chromatin, particularly lamin. Mutations in genes encoding LINC complex proteins can modulate transcription factor expression or tissue-specific gene patterns. Laminopathies exhibit ERK pathway hyperactivation, observed in *LMNA* mutations causing cardiomyopathy or SMAD6 overexpression, accelerating myogenic differentiation in *LMNA*-mutated cells [27, 72].

Nesprin-1 mutations primarily disrupt nuclear morphology by displacing lamin A/C, emerin, and SUN2 from the nuclear membrane, consequently disrupting the LINC complex. These mutations interfere with microtubule-driven protein interactions, leading to the disassembly of nucleus-microtubule connections [25, 85, 88, 100, 101]. In lower organisms like *Caenorhabditis elegans*, *ANC-1* mutations similarly result in misplacement of nuclei [72, 102]. Additionally, Nesprin-1 mutations in mammalian myoblasts impede the nuclear envelope recruitment of Akap450 during muscle cell differentiation [97, 98]. Mutations in *SYNE1/2* disrupt muscle-specific Nesprin isoforms, affecting nuclear morphology and nucleocytoskeletal coupling. These mutations potentially alter chromatin structural integrity, influencing cell signaling and gene regulation, including MAPK hyperactivation, perturbation of myogenic regulatory factor (MRF) expression, and alteration of mechanosensitive genes in Nesprin-1/2 double knockout (DKO) mouse cardiomyocytes [97].

Nesprin mutations exert a profound influence on cardiac structural and functional integrity, illuminating their pivotal role in cardiac pathophysiology. Recent investigations offer a comprehensive overview of the repercussions of Nesprin mutations on cardiac structure, particularly within the *SYNE1* gene [25]. Significantly, in-depth analyses highlight the functional implications, including compromised mechanical stability and disturbance of cellular architecture in cardiac myocytes. These mutations disrupt cytoskeletal organization, thereby impacting heart contractile function [27, 103]. By disrupting nuclear morphology and mechanical integrity, these mutations provide valuable insights into the pathophysiology of cardiac disorders.

## Advancing therapeutic strategies for nesprin-related muscular disorders

### Rectification of Nesprin gene mutations

Nesprin gene mutations underlie specific muscular degenerative diseases such as DCM and EDMD [15], offering potential avenues for corrective interventions through gene therapy. A range of techniques, including CRISPR/Cas9 gene editing [104], gene replacement therapy, RNA interference, antisense oligonucleotides, and homologous recombination repair, provides opportunities to rectify or substitute defective Nesprin genes at the cellular level [37, 72, 103, 105]. Despite promising advancements, these methodologies pose intricate biological and technical challenges. Many gene therapy and editing strategies, although evolving rapidly, currently remain primarily experimental, necessitating further extensive research and meticulous clinical trials to firmly establish their safety and efficacy.

### Controlling nesprin protein expression

Modulating Nesprin protein expression through pharmacological [106] or gene-based interventions holds promise in restoring or enhancing cardiac muscle cell functionality. These strategies encompass a spectrum of methodologies, including gene editing, RNA interference (RNAi), antisense oligonucleotides, transcription factor modulation, epigenetic regulation, and control over mRNA stability and protein degradation [107, 108]. Evaluating potential side effects and the broader repercussions of modulating protein expression on cellular mechanisms is paramount, especially in clinical applications where comprehensive assessments of safety and efficacy are pivotal. Given the critical role of Nesprin proteins in maintaining cellular structure and function, manipulating their expression could intricately impact diverse cellular processes. Thus, precise management of these interventions is essential to mitigate unintended cellular responses. For instance, in cases where decreased Nesprin expression correlates with specific cardiac conditions, the development of pharmaceuticals to boost its expression could offer valuable therapeutic benefits [107, 109].

### Enhancing LINC complex functionality

Recent studies have unveiled the potential therapeutic implications of targeting the LINC complex in LMNA-related EDMD and DCM [16, 110, 111]. By modulating the expression of Nesprin proteins or implementing gene therapy strategies, researchers aim to restore the structural integrity of cardiac muscle cells and enhance mechanotransduction capacity [16]. Additionally, pharmacological interventions targeting the components of the LINC complex offer promising avenues for therapeutic intervention [111]. Furthermore, refining mechanotransduction mechanisms, advancing cell therapy approaches, and exploring protein interactions within the LINC complex represent additional strategies to improve therapeutic outcomes for LMNA-related EDMD and DCM [110].

Nesprin serves as a pivotal component of the LINC complex, collaborating with SUN domain proteins, lamin A/C, and emerin at the nuclear membrane [15, 38, 39]. Enhancing the functionality of the LINC complex presents an opportunity to restore structural integrity and bolster mechanotransduction capacity within muscle cells [112–114]. This augmentation holds promise in potentially mitigating the progression of muscle degenerative diseases, thereby offering avenues for improved management and treatment outcomes for these conditions. The disruption of the LINC complex offers a novel therapeutic avenue with significant potential benefits for individuals with LMNA-related EDMD and DCM. Through targeted interventions aimed at restoring the integrity of the LINC complex, researchers are positioned to inaugurate a new era of precision medicine for muscular disorders, promising enhanced patient outcomes and quality of life.

### Augmenting cardiac muscle cell function

The focal point lies in amplifying the pivotal role of Nesprin protein in fortifying the connection between the nucleus and the cytoskeletal framework, essential for maintaining the structural stability of cardiac muscle cells. This endeavor encompasses a spectrum of strategies, ranging from modulating Nesprin protein expression to implementing gene therapy, pharmacological interventions, refining mechanotransduction mechanisms [96], advancing cell therapy, exploring protein interactions, and employing anti-inflammatory and antioxidant methodologies [106], alongside biomechanical interventions. Given the intricate nature of cardiac muscle cells and the multifaceted nature of heart diseases, the development of targeted small molecule drugs tailored specifically for the Nesprin protein emerges as a promising avenue toward potentially enhancing the functionality of cardiac muscle cells and fortifying overall cardiac performance.

## Conclusion

Nesprin, together with its associated components, stands as a critical determinant of nuclear stability and functional efficiency, essential for the preservation of normal muscle physiology and the prevention of muscle-related pathologies. As ongoing research penetrates deeper into unraveling the complex architecture and diverse functionalities of these protein complexes, a vast potential emerges for the discovery of novel therapeutic avenues and targets. This advancement holds immense promise in effectively tackling genetic muscle disorders, including DCM and EDMD. By further elucidating the intricate roles of these proteins, new pathways are illuminated, paving the way for significant breakthroughs in the management and treatment of such debilitating conditions, thereby offering renewed hope for improved patient outcomes in the foreseeable future.

### Supplementary Information

**Supplementary Material 1.**

## Data Availability

No datasets were generated or analysed during the current study.
