# Supplementary material for: Nesprin proteins: bridging nuclear envelope dynamics to muscular dysfunction
Source: Cell Commun Signal. 2024 Apr 2;22:208. doi: 10.1186/s12964-024-01593-y (PMC10986154; doi:10.1186/s12964-024-01593-y)
Supplement: Supplementary file 1 — Supplementary Material 1. [file 12964_2024_1593_MOESM1_ESM.docx]

Supplementary data Table 1

Tissue-specific Expression Profiles of Nesprin Isoforms

| Gene  Tissue | Nesprin-1G | Nesprin-1α1 | Nesprin-1α2 | Nesprin-1β1 | Nesprin-1β2 | Nesprin-2G | Nesprin-2α1 | Nesprin-2α2 | Nesprin-2β1 | Nesprin-2β2 | Nesprin-2ε1 | Nesprin-2ε2 | Nesprin-3 | Nesprin-4 |
| --- | --- | --- | --- | --- | --- | --- | --- | --- | --- | --- | --- | --- | --- | --- |
| Adipose | ++ | UN | + | + | + | +++ | + | +/- | UN | + | + | + | +++ | + |
| Bladder | + | UN | +/- | + | + | + | +/- | + | UN | + | +/- | + | UN | + |
| Brain | ++ | UN | +/- | + | + | + | +/- | +/- | UN | +/- | +/- | + | +/- | + |
| Cervix | ++ | UN | +/- | + | + | ++ | +/- | + | UN | + | + | + | UN | + |
| Colon | ++ | UN | + | + | + | + | +/- | + | UN | + | + | + | + | + |
| Esophagus | + | UN | + | + | + | ++ | + | + | UN | + | + | UN | UN | + |
| Heart | ++ | UN | +++ | + | + | + | +++ | + | UN | +/- | +/- | ++ | +/- | + |
| Kidney | ++ | UN | + | + | + | +++ | +/- | + | UN | + | + | + | ++ | + |
| Liver | + | UN | + | + | + | +++ | + | + | UN | + | + | + | + | + |
| Lung | ++ | UN | + | + | + | ++ | +/- | + | UN | + | + | + | +/- | + |
| Ovary | +++ | UN | +/- | + | + | +++ | + | + | UN | +/- | + | + | + | + |
| Placenta | + | UN | + | + | + | +++ | +/- | + | UN | + | + | + | UN | + |
| Prostate | ++ | UN | +/- | + | + | ++ | + | + | UN | + | + | + | UN | + |
| Skeletal muscle | + | UN | +++ | + | + | +++ | +++ | +/- | UN | + | + | ++ | + | + |
| Small intestine | + | UN | +/- | + | + | ++ | +/- | + | UN | + | + | + | UN | + |
| Spleen | ++ | UN | + | ++ | + | + | +/- | + | UN | + | + | + | ++ | + |
| Testis | ++ | UN | +/- | + | + | ++ | + | + | UN | + | + | + | UN | + |
| Thymus | + | UN | +/- | +/- | + | + | +/- | +/- | UN | + | +/- | + | UN | + |
| Thyroid | +++ | UN | +/- | + | + | +++ | + | + | UN | + | + | + | UN | + |
| Trachea | ++ | UN | + | + | + | +++ | + | + | UN | + | + | + | UN | + |
| ESC | +/- | UN | UN | +/- | +/- | + | UN | + | UN | +/- | + | +/- | + | UN |
| NHF | + | UN | +/- | +/- | +/- | + | UN | + | UN | +/- | +/- | +/- | UN | UN |
| LCL | + | UN | +/- | +/- | +/- | + | +/- | + | UN | + | +/- | +/- | UN | UN |
| Ntera-2 | +/- | UN | +/- | UN | +/- | + | +/- | + | UN | +/- | + | +/- | UN | UN |
| HeLa | +/- | UN | +/- | +/- | +/- | + | +/- | + | UN | +/- | +/- | UN | UN | UN |
| U2OS | + | UN | UN | +/- | +/- | + | UN | +/- | UN | +/- | +/- | UN | UN | UN |
| VSMC | ++ | UN | +/- | + | + | + | UN | UN | UN | +/- | +/- | UN | UN | + |

1.+++:indicates isoforms that are prominently expressed in specific tissues, playing a critical role in the tissue's function or development.

2.++:isoforms that isoforms were more expressed in this tissue, but lacked obvious tissue specificity.

3.+:signifies isoforms that have minimal to expression in certain tissues, suggesting these isoforms do not play a significant role in those tissue types.

4.+/-:expression levels imply the isoform is present in such minimal amounts that its biological significance in those tissues remains uncertain or indicates a highly specialized function.

5.UN:It was not possible to determine the efficiency of amplification.
